# Supplementary material for: A Ubl/ubiquitin switch in the activation of Parkin
Source: EMBO J. 2015 Aug 7;34(20):2492–505. doi: 10.15252/embj.201592237 (PMC4609182; doi:10.15252/embj.201592237)
Supplement: Supplementary file 1 — Expanded View Figures PDF [file embj0034-2492-sd1.pdf]

Expanded View Figures

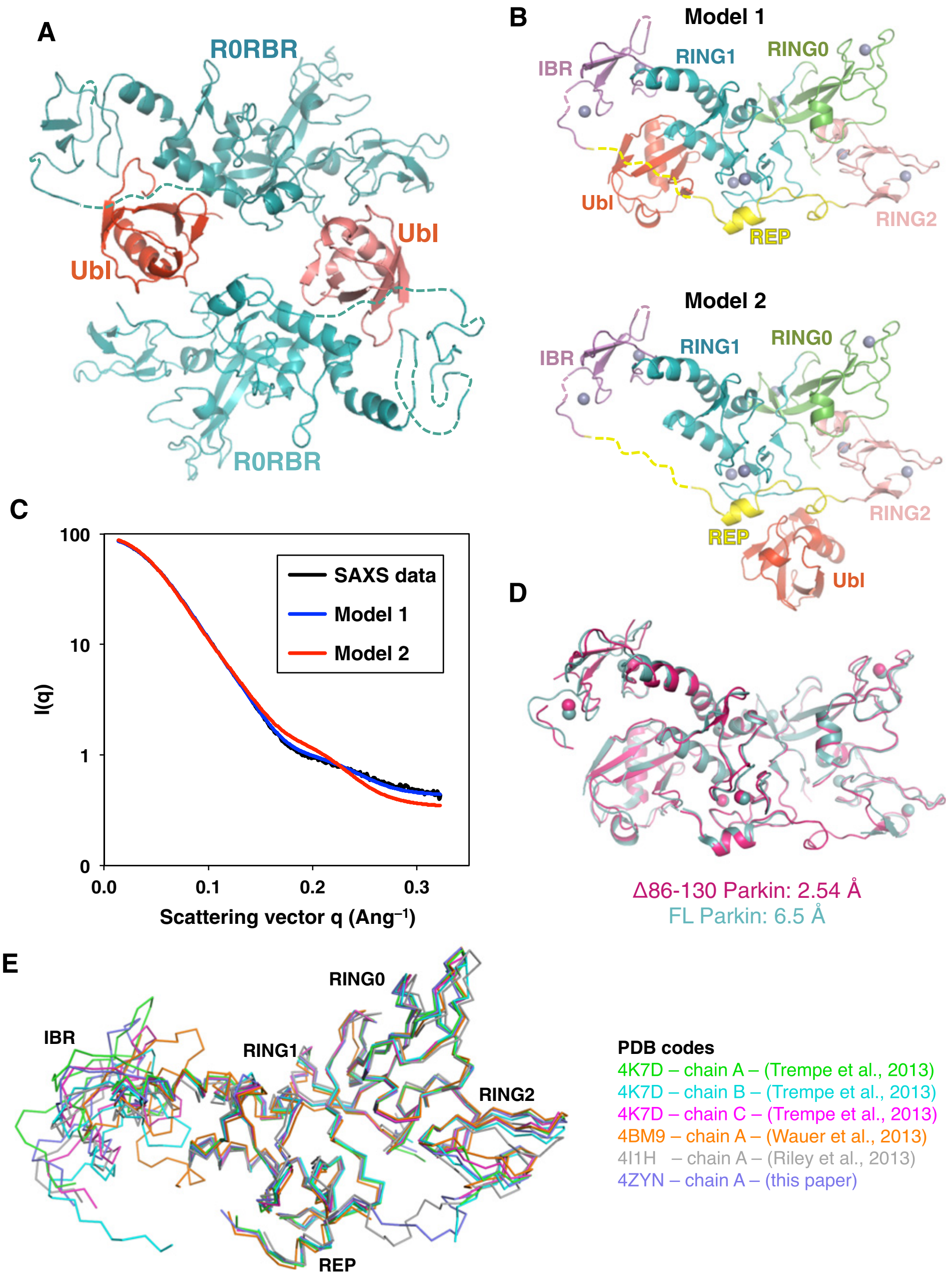

Figure EV1. Structural details of Ubl binding and IBR flexibility.

A Two molecules of Δ86–130 Parkin in the asymmetric unit. The density for the linker between the Ubl domain (red) and R0RBR (cyan) is absent, leading to two alternative models for the position of the Ubl domain.

B Models of the Ubl domain bound to the R0RBR module.

C Solution SAXS data of Parkin Δ86–130 (black) superimposed with the scattering curves calculated from the two models shown in (B). Only model 1 fits the experimental scattering data, with a χ^2 of 2.5, compared to 57.8 for model 2.

D Overlay of the high-resolution Parkin Δ86–130 structure with the previous low-resolution structure of full-length Parkin (Trempe *et al*, 2013). Despite very different packing and crystal contacts, the two structures are essentially identical. The main differences in the Δ86–130 structure are greater mobility (and disorder) in the IBR domain and the ordering of the REP-RING2 linker.

E Overlay of different Parkin structures, showing the different positions adopted by the IBR domain. The Ubl domain was omitted for clarity of presentation.

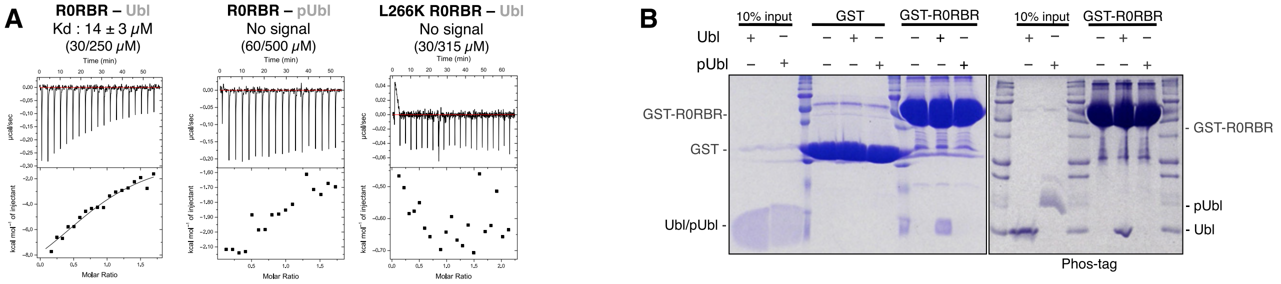

C

Parkin construct	Rg	Estimated Mass (kDa)	
		Porod	Qr
WT Parkin	29.1 ± 0.1	54.4	48.9 ± 0.4
L266K Parkin	34.1 ± 0.4	53.6	44.6 ± 1.1
Δ86-130 Parkin	27.0 ± 0.1	45.5	39.8 ± 0.4
pParkin	31.9 ± 0.3	50.5	45.2 ± 0.8
pParkin + pUb	33.1 ± 0.3	54.7	55.0 ± 1.3
W403A Parkin	30.1 ± 0.1	55.6	50.8 ± 0.4

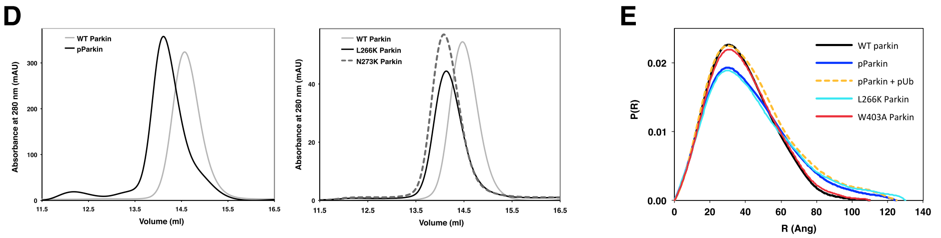

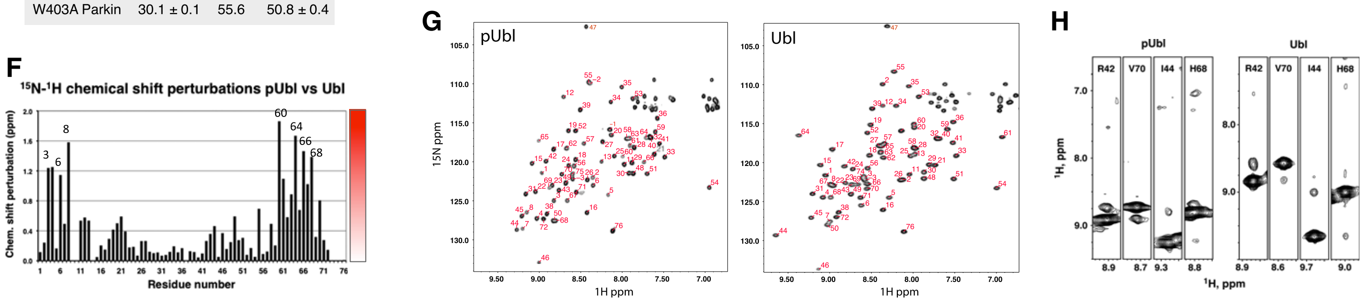

Figure EV2. Conformational changes associated with Ubl phosphorylation.

A Repeat of the ITC measurement with a different concentration of the isolated Parkin Ubl and R0RBR module. The experiments with phosphorylated Ubl and L266K from Fig 2A are replotted for comparison.

B Pull down of free Ubl or pUbl using GST-Parkin-R0RBR constructs immobilized on glutathione–Sepharose resin. After washes, products were resolved using standard or Phos-tag SDS–PAGE.

C SAXS-derived parameters for different Parkin constructs.

D Size-exclusion chromatography of various Parkin constructs. Concentrated protein solutions were injected on a Superdex 200 Increase 10/300 GL column at 1 ml/min, equilibrated in SAXS buffer.

E Pair-distance distribution functions derived from SAXS curves for different Parkin constructs.

F Backbone amide NMR chemical shifts perturbations induced by Ser65 phosphorylation in the Ubl domain of Parkin.

G ^{15}N-HSQC NMR spectra of ^{15}N-labeled Parkin pUbl and Ubl.

H Comparison of strips from ^{15}N-NOESY-HSQC experiments on ^{15}N-labeled pUbl and Ubl. The H^N-H^N cross-peaks between the pairs of residues Arg42-Val70 and Ile44-His68 located around the C-terminal β-strand are identical.

 © 2015 The Authors

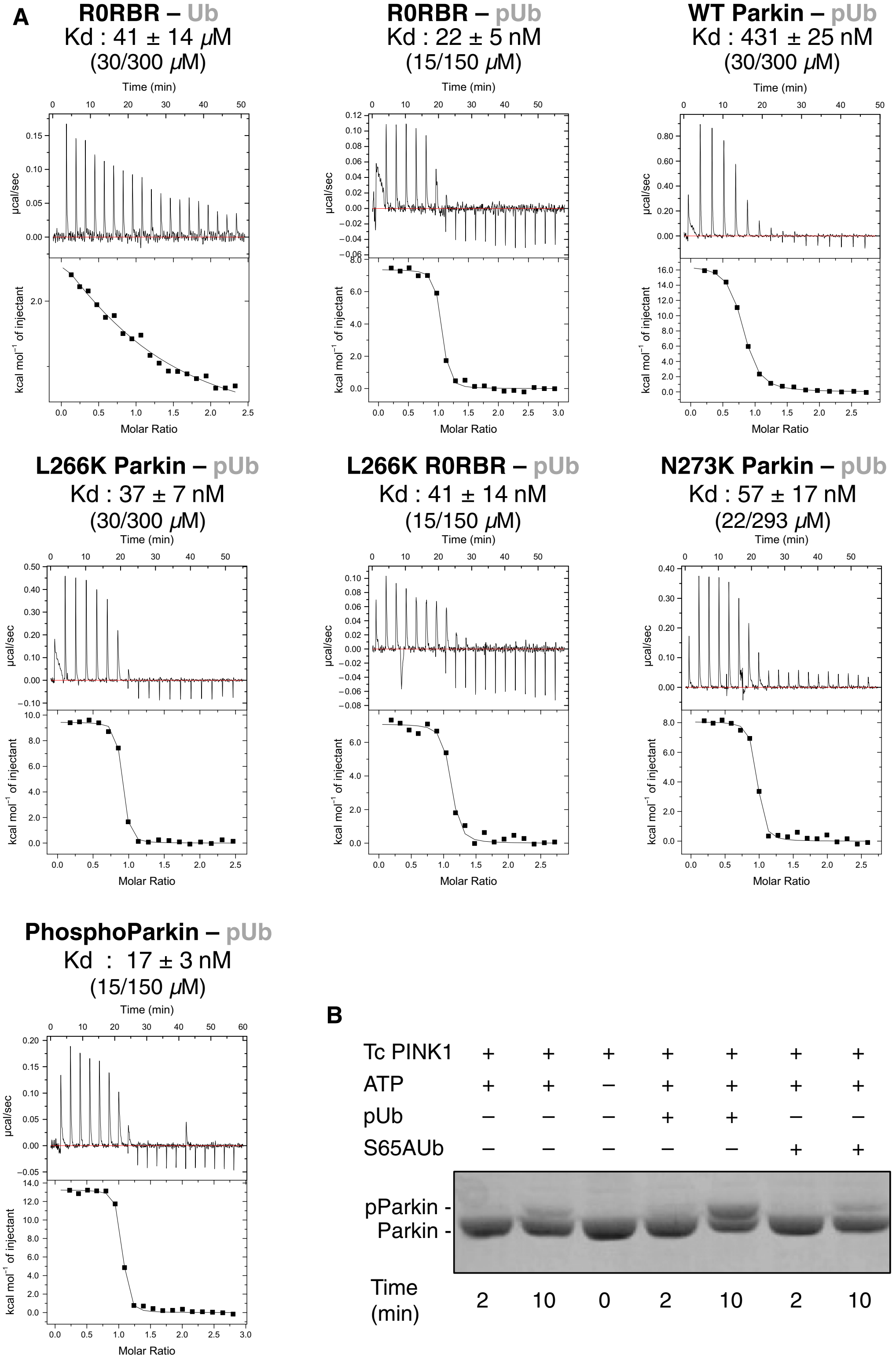

Figure EV3. ITC data and additional Parkin phosphorylation assay.

A ITC data and fits for the titration of different Parkin constructs with pUb shown in the table in Fig 3A.

B Parkin phosphorylation assay showing that unphosphorylated ubiquitin does not stimulate Parkin phosphorylation by PINK1. The S65A mutant of ubiquitin was used to prevent the formation of pUb during the assay. His-tagged ubiquitin was used in this assay.

© 2015 The Authors

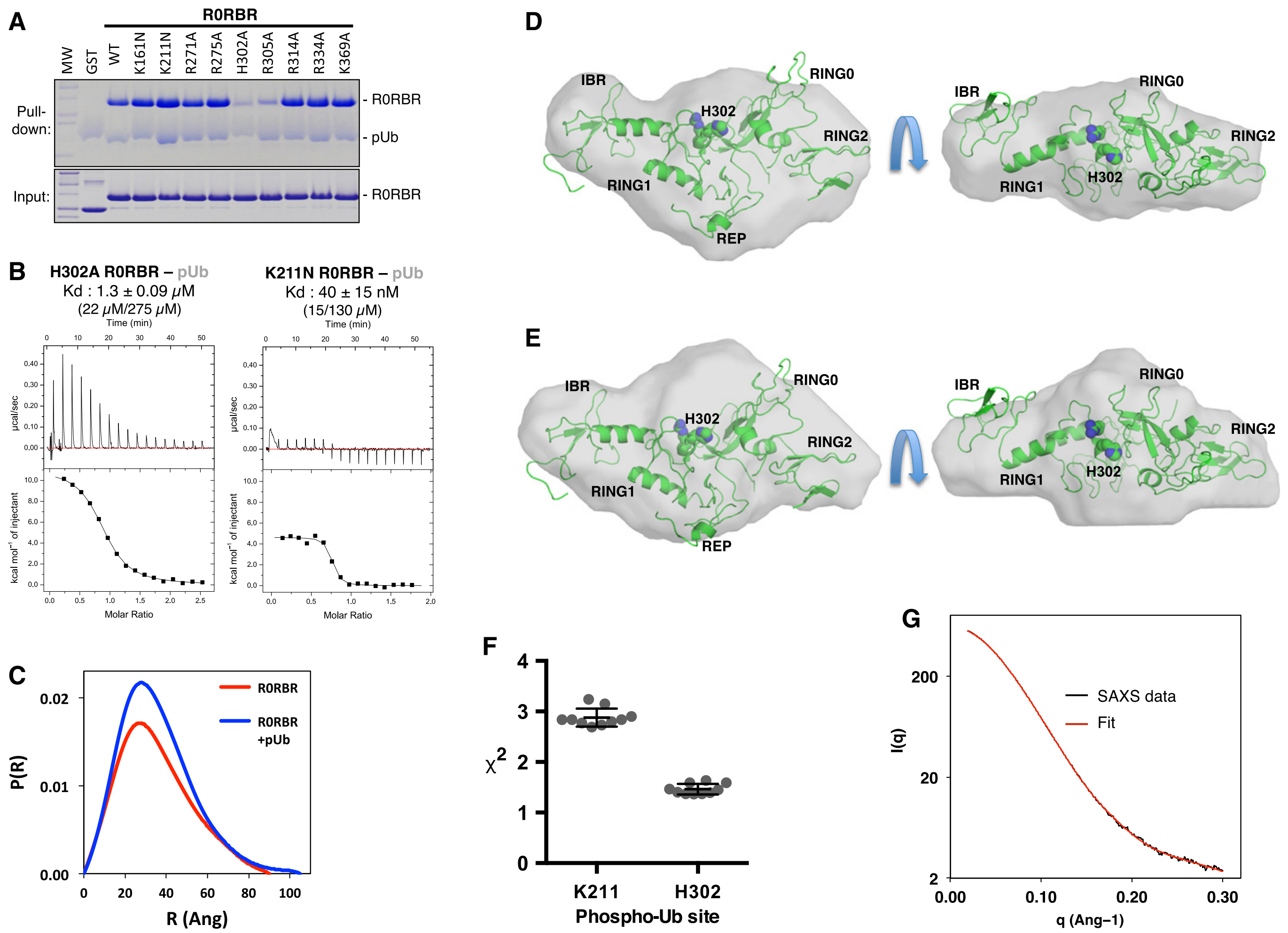

Figure EV4. SAXS modeling of the complex of pUb and Parkin.

A Replicate of the pull down of Parkin R0RBR mutants using His-tagged pUb, immobilized on Ni-NTA agarose. Bound proteins were eluted, resolved by SDS–PAGE, and stained with Coomassie Blue.

B ITC data and fits for the titration of two different Parkin R0RBR mutants, representing the two major sulfate-binding sites, with pUb. The results are summarized in the table in Fig 4B.

C Pair-distance distribution functions derived from SAXS curves for R0RBR and the R0RBR–pUb complex.

D *Ab initio* shape determination for R0RBR (light gray surface), superposed with the R0RBR structure (green). The image on the right is a 90° rotation around the horizontal axis.

E *Ab initio* shape determination for the R0RBR–pUb complex (light gray surface), superposed with the R0RBR structure (green).

F Chi-square values for the ten best R0RBR–pUb SAXS docking models for two potential phospho-Ser65 interaction sites. Horizontal bars indicate average and standard deviation.

G Overlay of the experimental SAXS data from the R0RBR–pUb complex (black) and the calculated scattering curve from the best rigid-body model (red).

 © 2015 The Authors

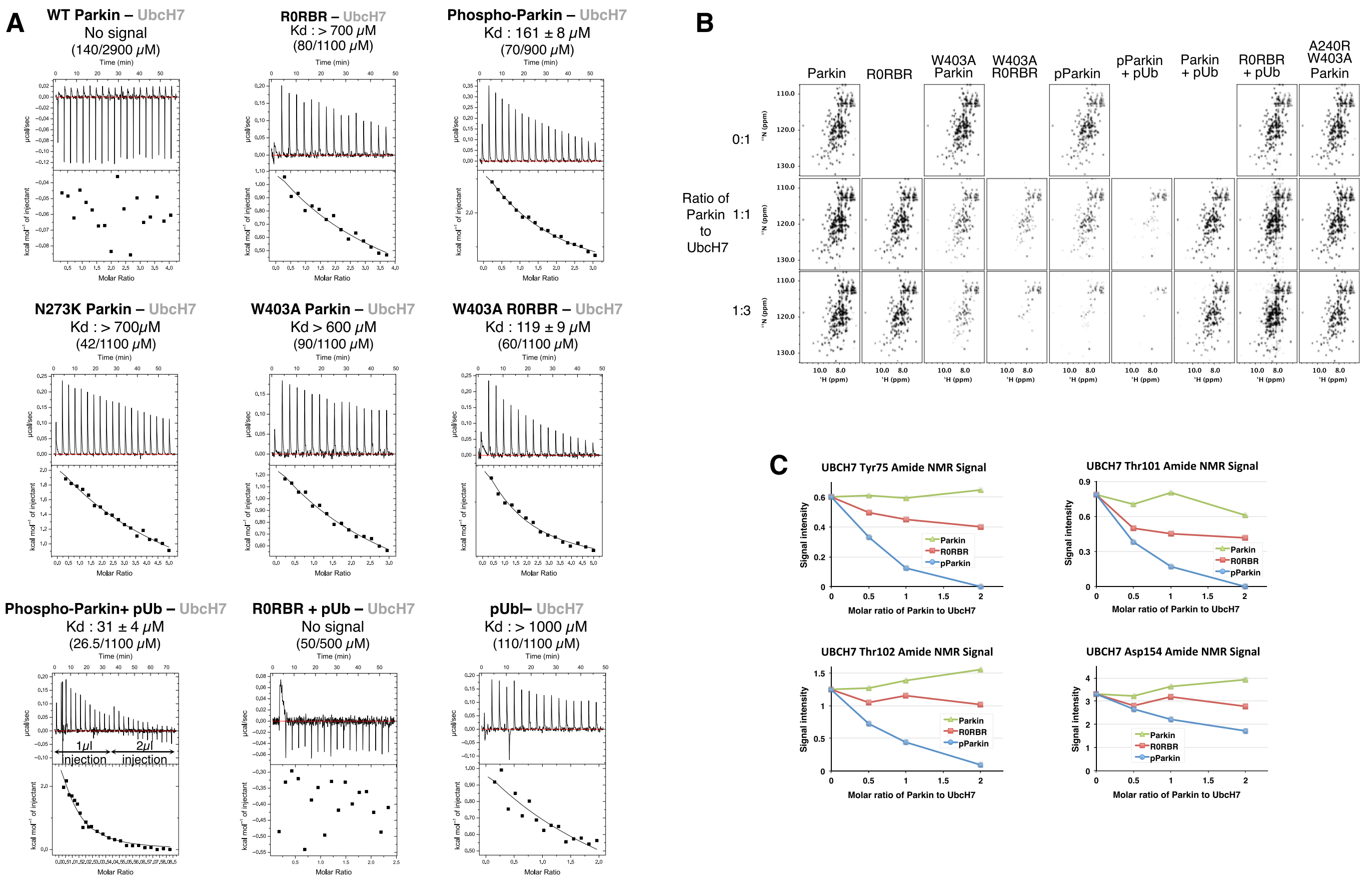

Figure EV5. ITC and NMR measurements of UbcH7 binding to Parkin.

A ITC data and fits for the titration of different Parkin constructs with UbcH7. The results are tabulated in Fig 5B.

B NMR analysis of Parkin binding to ^{15}N-labeled UbcH7. The UbcH7 and Parkin concentrations were 1:0 (200 μM), 1:1 (120 μM:120 μM), and 1:3 (65 μM:195 μM). Spectra were adjusted to account for the number of transients and the concentration of ^{15}N-UbcH7. Reference spectra for free ^{15}N-UbcH7 are displayed above titrations with the Parkin constructs. The spectra in Fig 5C are enlargements of the 1:1 titration spectra.

C Signal intensity of ^{15}N-UbcH7 amides as a function of addition of Parkin, R0RBR, and pParkin. Conditions were the same as in Fig 5D.

© 2015 The Authors
